# Residue-Ligand Interaction Energy (ReLIE) on a Receptor-Dependent 3D-QSAR Analysis of S- and NH-DABOs as Non-Nucleoside Reverse Transcriptase Inhibitors

**DOI:** 10.3390/molecules17077666

**Published:** 2012-06-25

**Authors:** Monique Araújo de Brito, Carlos Rangel Rodrigues, José Jair Viana Cirino, Jocley Queiroz Araújo, Thiago Honório, Lúcio Mendes Cabral, Ricardo Bicca de Alencastro, Helena Carla Castro, Magaly Girão Albuquerque

**Affiliations:** 1 Laboratory of Computational Medicinal Chemistry (LabQMC), Faculty of Pharmacy, Fluminense Federal University (UFF), Niterói, RJ, 24241-000, Brazil; 2 Laboratory of Molecular Modeling (LabMMol), Program of Post-Graduation in Chemistry (PPGQu), Institute of Chemistry, Federal University of Rio de Janeiro (UFRJ), Rio de Janeiro, RJ, 21941-909, Brazil; 3 Laboratory of Molecular Modeling & QSAR (ModMolQSAR), Faculty of Pharmacy, Federal University of Rio de Janeiro (UFRJ), Rio de Janeiro, RJ, 21941-590, Brazil; 4 Laboratory of Antibiotics, Biochemistry, Education and Molecular Modeling (LABiEMol), Institute of Biology, Fluminense Federal University (UFF), Niterói, RJ, 24210-130, Brazil

**Keywords:** receptor-dependent 3D-QSAR, residue-ligand interaction energy, molecular dynamics, DABO derivatives, reverse transcriptase, AIDS/HIV-1

## Abstract

A series of 74 dihydroalkoxybenzyloxopyrimidines (DABOs), a class of highly potent non-nucleoside reverse transcriptase inhibitors (NNRTIs), was retrieved from the literature and studied by receptor-dependent (RD) three-dimensional quantitative structure-activity relationship (3D-QSAR) analysis to derive RD-3D-QSAR models. The descriptors in this new method are the steric and electrostatic interaction energies of the protein-ligand complexes (per residue) simulated by molecular dynamics, an approach named *Residue-Ligand Interaction Energy* (ReLIE). This study was performed using a training set of 59 compounds and the MKC-442/RT complex structure as reference. The ReLIE-3D-QSAR models were constructed and evaluated by genetic algorithm (GA) and partial least squares (PLS). In the best equations, at least one term is related to one of the amino acid residues of the p51 subunit: *Asn136*, *Asn137*, *Glu138*, and *Thr139*. This fact implies the importance of interchain interaction (p66-p51) in the equations that best describe the structure-activity relationship for this class of compounds. The best equation shows q^2^ = 0.660, SE_cv_ = 0.500, r^2^ = 0.930, and SEE = 0.226. The external predictive ability of this best model was evaluated using a test set of 15 compounds. In order to design more potent DABO analogues as anti-HIV/AIDS agents, substituents capable of interactions with residues like *Ile94*, *Lys101*, *Tyr181*, and *Tyr188* should be selected. Also, given the importance of the conserved *Asn136*, this residue could become an attractive target for the design of novel NNRTIs with improved potency and increased ability to avoid the development of drug-resistant viruses.

## 1. Introduction

The human immunodeficiency virus (HIV) is the etiological agent of the acquired immunodeficiency syndrome (AIDS). There are two HIV species: HIV-1 (of high virulence/infectivity and global prevalence) and HIV-2 (of low virulence/infectivity and prevalent on West Africa). The HIV is a retrovirus distinguished by the presence of a viral reverse transcriptase (RT), among other targets such as protease and integrase, responsible for the synthesis of DNA from the viral RNA genome [1].

Due to its essential role in the replication of the virus, this enzyme is one of the most important antiviral targets in the chemotherapy of AIDS [2]. The RT enzyme is a heterodimer, consisting of p66 and p51 subunits, the latter being a truncated form of the former [2]. Although each subunit consists of thumb, palm, and finger domains, only the p66 subunit contains a functional active site that binds the nucleic acid template-primer to the nucleoside triphosphates [2].

There are two classes of antiretroviral drugs currently used to treat AIDS that target the HIV-RT: nucleoside/nucleotide analog RT inhibitors (NRTIs) and non-nucleoside RT inhibitors (NNRTIs) [3,4]. The NRTIs (e.g., AZT, ddI, ddC, and d4T) are HIV-1/HIV-2 RT competitive substrate inhibitors that bind to the active site, and can be incorporated into the growing DNA chain. Further elongation, however, is not possible, as they lack the 3'-OH group present in the natural substrate, which causes premature termination of the growing viral DNA strand [5].

**Figure 1:**
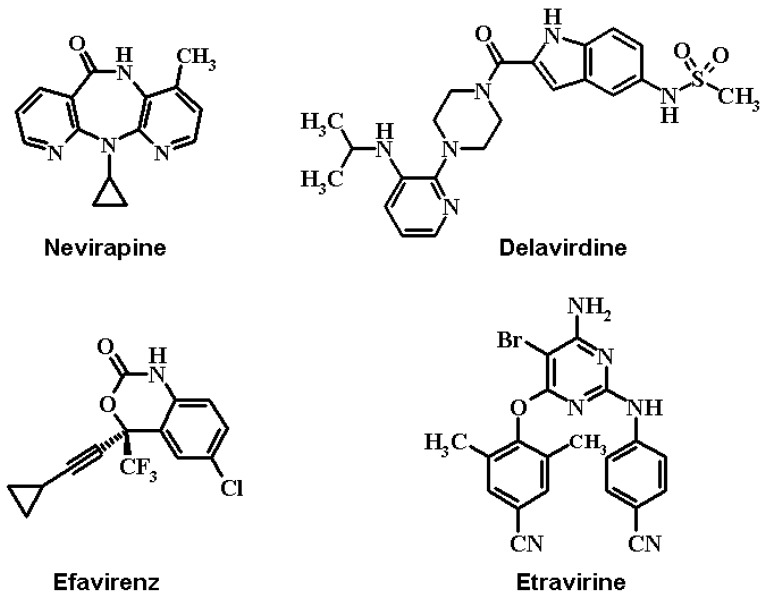
Structures of the four non-nucleoside reverse transcriptase inhibitors (NNRTIs), nevirapine, delavirdine, efavirenz, and etravirine, approved by the US Food and Drug Administration (FDA) to treat AIDS.

In contrast, NNRTIs (e.g., nevirapine, delavirdine, efavirenz, and etravirine, Figure 1) [6,7] are selective HIV-1 RT non-competitive inhibitors that bind to an allosteric site (non-nucleoside binding site, NNBS), which is located in the p66 subunit, about 10 Å from the active site [4,5]. Thus, these inhibitors also impair the DNA synthesis process.

Binding of NNRTIs is accompanied by dramatic rearrangements of the subdomains, indirectly influencing the enzyme catalytic efficiency. The most prominent change seen is in the position of the thumb domain, which is locked in an upright conformation upon NNRTI binding. Moreover, the NNRTI binding deforms the sheet of the p66 palm subdomain, affecting the precise positioning of the primer strand relative to the polymerase active site [7]. 

Comparisons of the structures of the free and inhibitor bound enzymes show that NNRTIs also modify the position of the three catalytic residues (Asp110, Asp185, and Asp186) relative to the other active site residues [5]. High-resolution crystal structures of the HIV-1 RT unbound and in complex with nevirapine, delavirdine, efavirenz, or etravirine inhibitors show that all of these compounds bind to the same allosteric site, even though their 2D structures are quite different [2,4,5,6,8,9].

### 1.1. Resistance of HIV-1 to NNRTIs

HIV has a high rate of replication, which can reach up to 10^10^ viral particles per day in an untreated individual [10]. Additionally, in the process of the virus reverse transcription, there are a high number of mutations, estimated in the order of 10^4^ to 10^5^ times per day [11]. The high rate of mutation combined with the consequent genetic variability has as its most important consequence the selection and the predominance of strains resistant to anti-HIV drugs currently used to treat AIDS.

The emergence of viral variants resistant to drugs in patients infected with HIV is the main cause of failure in treatment [3,12,13]. The development of resistance is an important factor when considering the administration of a drug for a prolonged period [13]. In this case, the inhibitor becomes a part of a selective pressure for the survival of the virus, and the process of mutation becomes accelerated according to the misuse of anti-HIV drugs [13].

The mutations alter the affinity of the RT inhibitors, resulting usually in decrease of van der Waals interactions between enzyme and inhibitor [12,14,15,16,17,18,19]. The development of resistance is a significant problem in the class of NNRTIs [12,13,15]. Simple changes such as Leu100Ile, Lys101Glu, Lys103Asn, Val106Ala, Val108Ile, Tyr181Cys, Tyr188Leu, Gly190Ala, Pro225His, and Phe227Leu [14,15,20,21,22,23,24,25], and double mutations such as Lys103Asn/Tyr181Cys [22,26], Lys103Asn/Val108Ile, Lys103Asn/Pro225His, and Lys103Asn/Leu100Ile [24] have been described in the literature as a function of prolonged use of NNRTIs [12].

In order to overcome resistance, a successful AIDS treatment regimen, known as highly active antiretroviral therapy (HAART) [27], is in current use, which combines three drugs: two NRTIs plus a NNRTI or a PI (protease inhibitor). Therefore, there is urgent need for the development of new HIV-1 enzyme (e.g., RT, protease, integrase) inhibitors in order to overcome this pandemic disease.

### 1.2. Three-Dimensional Quantitative Structure-Activity Relationship Studies

A 3D-QSAR model is a mathematical expression that relates the variation of the biological response in a series of compounds to the variation in their 3D chemical structure [28,29]. The relation between the spatial interactions (independent variables) and the biological response (dependent variable) can be established by use of the partial least-squares (PLS) regression method [30,31,32], which is becoming the statistical method of choice for most QSAR studies [33,34,35,36].

By careful selection of the biological data set (the training and test set compounds) and careful model construction (e.g., the trial alignment and the putative active conformation), a 3D-QSAR study can lead to a useful model that could be used to predict the biological activity values of new compounds prior to their synthesis, which is the primary goal of any drug design process in the medicinal chemistry field [29,30,37,38,39,40,41].

Molecular modeling approaches currently used in Computer-Aided/Assisted Drug Design (CADD) are classified as: (i) *direct*, *receptor-based*, or *structure-based*, which depend on the receptor geometry; and (ii) *indirect* or *ligand-based*, which do not depend on the knowledge of the receptor geometry [29]. Hopfinger has proposed a similar classification for the 3D-QSAR approaches as *Receptor Dependent* (RD) and *Receptor Independent* (RI) methods [42,43]. Therefore, RD-3D-QSAR models are derived from the 3D structure of the receptor-ligand complex, while the RI-3D-QSAR models are derived from the 3D structure of the ligands. The RI-3D-QSAR approach is the more usual case, and a typical example is the CoMFA method [44]. The RD-3D-QSAR approach is a less usual case, and as example there are COMBINE [45,46] and RD-4D-QSAR methods [42].

The Genetic Algorithm (GA) is a particularly useful technique in solving problems with a large number of variables, by allowing an efficient sampling of the available solutions [47,48,49]. GAs have been applied to various molecular modeling problems in drug design, such as conformation/orientation searches (essential in the docking method); studies of SAR (which help the search for pharmacophores) and QSAR (which help the correlation of descriptors with biological activities) [30,48,50].

In a QSAR study using GAs, the models are randomly created and those with better statistical values propagate their characteristics *(genetic material*) by crossover operations, which is a combination of independent variables of two good models (*parents*) to create a new model (*child*) [47,51,52]. In the next generation, the models with best scores are kept and new models are created by crossover and mutation operations. The mutation is the creation of a new model by the random introduction of a new variable in the model created by crossover, which helps maintain sufficient diversity in the population [47,49].

Genetic Function Approximation (GFA) is a GA technique used to create QSAR models, where the variables are called base functions [53,54]. GFA applies the same procedures described above for GAs, and coupled with PLS, the GFA-PLS technique has as its most important feature the generation of multiple good models rather than the optimization of only a single model [53]. Several authors have reported the use of combined GA and PLS analyses [28,55,56,57,58,59,60,61].

Recently, we reported a RI-3D-QSAR model (CoMFA) [62], using a series of 74 S- and NH-DABO (dihydroalkoxybenzyloxopyrimidine) HIV-1 NNRT inhibitors [63,64,65,66], selected as an unprecedented series in 3D-QSAR studies. Now, to complement this study and to add more information to the SAR study of this class of NNRTIs, we have constructed and evaluated RD-3D-QSAR models by GFA-PLS method, using as descriptors the steric and electrostatic interaction energies of the protein-ligand complexes (*per residue*) simulated by molecular dynamics (MD), a new approach named *Residue-Ligand Interaction Energy* (ReLIE). Those models may prove to be useful in understanding the most relevant residues for DABOs interaction and, consequently, in designing new non-nucleoside RT inhibitors for the AIDS treatment.

### 1.3. Computational Approach

#### 1.3.1. Structural and Biological Database

The biological activity of the 74 compounds selected from the literature [63,64,65,66] for this study was evaluated *in vitro* against the HIV-1 RT enzyme, according to the same pharmacological protocol [66]. The inhibitory potencies, given in IC_50_ (μM), were transformed into pIC_50_ (M), which corresponds to the logarithm of the inverse of the minimum concentration capable of inhibiting 50% of enzyme activity. Table 1 shows the chemical structures and the biological activities of this series. The compounds containing a stereogenic center (*i.e.*, Y = *sec*-butyl, Table 1), corresponding thus to a racemate, were defined in absolute *R* configuration and its original values of IC_50_ were multiplied by two. As this stereogenic center is located in an alkyl chain side, it was considered of lower importance and the *R* enantiomer was arbitrarily defined as the eutomer.

**Table 1:** Structures of the S- and NH-DABO derivatives and the corresponding HIV-1 RT inhibitory potencies (pIC_50_) [63,64,65,66]. 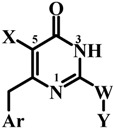

# ^a^	X ^b^	Ar	W–Y ^c^	pIC_50_^d^			# ^a^	X ^b^	Ar	W–Y ^c^	pIC_50_^d^
**1**	Me	2-naphtyl	S- *sec*-Bu	4.23			**38**	H	2,6-di-F-Ph	S-Me	6.10
**2**	H	1-naphtyl	S-cyclopentyl	4.31			**39**	Me	2-Cl-Ph	S- *sec*-Bu	6.10
**3**	Me	1-naphtyl	S-cyclopentyl	4.35			**40**	Me	2-F-Ph	S- *sec*-Bu	6.10
**4**	Me	4-F-Ph	S- *sec*-Bu	4.59			**41**	Me	3-NO_2_-Ph	S- *sec*-Bu	6.10
**5**	Me	4-Cl-Ph	S- *sec*-Bu	4.77			**42**	H	2-F-Ph	S- *sec*-Bu	6.22
**6**	H	1-naphtyl	S- *sec*-Bu	4.79			**43**	H	3-NO_2_-Ph	S- *sec*-Bu	6.22
**7**	H	2-naphtyl	S- *sec*-Bu	4.83			**44**	H	2,6-di-Cl-Ph	S- *tert*-Bu	6.22
**8**	H	4-F-Ph	S- *sec*-Bu	4.83			**45**	H	2,6-di-Cl-Ph	S- *n*-Bu	6.30
**9**	H	4-Cl-Ph	S- *sec*-Bu	5.02			**46**	H	2,6-di-Cl-Ph	S-cyclopentyl	6.40
**10**	H	Ph	S- *tert*-Bu	5.07			**47**	H	2,6-di-F-Ph	S- *n*-Bu	6.70
**11**	H	3-Me-Ph	S- *tert*-Bu	5.09			**48**	H	2,6-di-F-Ph	S- *tert*-Bu	6.70
**12**	Me	3-Me-Ph	S- *sec*-Bu	5.27			**49**	H	2,6-di-Cl-Ph	S- *sec*-Bu	6.70
**13**	Me	2,6-di-Cl-Ph	S-cyclohexyl	5.31			**50**	Me	2,6-di-Cl-Ph	S- *sec*-Bu	6.92
**14**	Me	Ph	S-Me	5.31			**51**	H	2,6-di-F-Ph	S- *sec*-Bu	7.00
**15**	Me	Ph	S- *sec*-Bu	5.32			**52**	Me	2,6-di-F-Ph	S- *sec*-Bu	7.00
**16**	Me	3-Me-Ph	S- *tert*-Bu	5.34			**53**	H	2,6-di-F-Ph	S-cyclohexyl	7.05
**17**	Me	Ph	S-cyclohexyl	5.37			**54**	Me	2,6-di-F-Ph	S- *tert*-Bu	7.05
**18**	H	3-Cl-Ph	S- *sec*-Bu	5.42			**55**	H	2,6-di-F-Ph	S-cyclopentyl	7.10
**19**	Me	4-NO_2_-Ph	S- *sec*-Bu	5.44			**56**	Me	2,6-di-F-Ph	S-cyclopentyl	7.10
**20**	Me	3-Me-Ph	S-cyclopentyl	5.47			**57**	H	2,6-di-F-Ph	NH-cyclopentyl	7.15
**21**	H	2-Cl-Ph	S- *sec*-Bu	5.49			**58**	H	2,6-di-F-Ph	S- *iso*-Pr	7.30
**22**	Me	3-F-Ph	S- *sec*-Bu	5.52			**59**	Me	2,6-di-F-Ph	NH-cyclopentyl	7.52
**23**	H	2,6-di-Cl-Ph	S-Me	5.52			**60**	Me	1-naphtyl	S- *sec*-Bu	4.35
**24**	H	Ph	S-cyclohexyl	5.52			**61**	H	2-naphtyl	S-cyclohexyl	4.48
**25**	H	3-Me-Ph	S- *iso*-Pr	5.54			**62**	H	Ph	S- *sec*-Bu	5.27
**26**	H	Ph	S-cyclopentyl	5.55			**63**	Me	Ph	S-cyclopentyl	5.47
**27**	H	3-Me-Ph	S-cyclohexyl	5.59			**64**	H	3-Me-Ph	S-cyclopentyl	5.59
**28**	Me	3-Me-Ph	S-Me	5.60			**65**	Me	Ph	S- *iso*-Pr	5.60
**29**	Me	3-Me-Ph	S- *iso*-Pr	5.60			**66**	H	3-Me-Ph	S- *sec*-Bu	5.62
**30**	H	4-NO_2_-Ph	S- *sec*-Bu	5.62			**67**	Me	3-Cl-Ph	S- *sec*-Bu	5.74
**31**	Me	3-Me-Ph	S-cyclohexyl	5.66			**68**	H	3-F-Ph	S- *sec*-Bu	5.92
**32**	Me	Ph	S- *tert*-Bu	5.72			**69**	H	2-NO_2_-Ph	S- *sec*-Bu	6.22
**33**	Me	2,6-di-Cl-Ph	S-cyclopentyl	5.80			**70**	H	2,6-di-Cl-Ph	S-cyclohexyl	6.40
**34**	H	2,6-di-Cl-Ph	S- *iso*-Pr	5.89			**71**	Me	2,6-di-F-Ph	S-Me	6.70
**35**	Me	2,6-di-Cl-Ph	S- *iso*-Pr	5.94			**72**	Me	2,6-di-F-Ph	S- *n*-Bu	7.05
**36**	Me	2,6-di-Cl-Ph	S- *n*-Bu	5.94			**73**	Me	2,6-di-F-Ph	S-cyclohexyl	7.15
**37**	Me	2,6-di-Cl-Ph	S- *tert*-Bu	5.96			**74**	Me	2,6-di-F-Ph	S- *iso*-Pr	7.30

^a^ Underlined numbers correspond to test set compounds (**60**–**74**); ^b^ DABOs pyrimidine nucleobase uracil (X=H) or thymine (X=Me); ^c^ S-DABO (W = S, Y = alkyl) and NH-DABO (W = NH; Y = alkyl) series; ^d^ The original IC_50_ values of compounds containing a stereogenic center (W = S, Y = *sec*-Bu) were multiplied by two and only the *R* isomers were considered in this study.

#### 1.3.2. Definition of the Training and Test Sets

The 74 inhibitors were divided into a training set, containing 59 compounds (**1**–**59**), and a test set, containing 15 compounds (**60**–**74**), representing about 20% of all compounds (Table 1). The overall distribution of biological activity values (pIC_50_) ranges from 4.23 to 7.52 M and from 4.35 to 7.30 M in the training and test sets, respectively. In both sets, the compounds are regularly distributed throughout the whole range of activity, which comprises about four logarithmic units, and have the same structural diversity.

#### 1.3.3. Construction and Optimization of the Ligands

**Figure 2:**
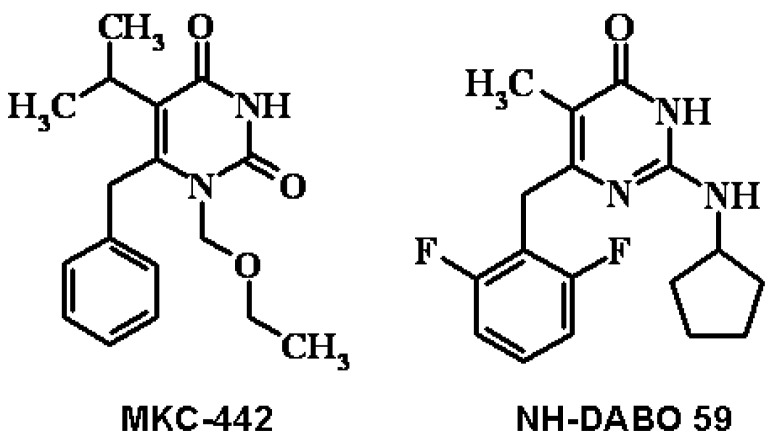
Structures of MKC-442 (template) and the most potent NH-DABO **59**.

In the absence of a DABO structure co-crystallized with the HIV-1 RT enzyme, the entire set of DABO derivatives (Table 1) were built according to the conformation of MKC-442 (or emivirine, Figure 2) bound to the HIV-1 RT (wild-type), available in the Protein Data Bank (PDB) [67] under code 1RT1 [68]. MKC-442, a NNRTI of the hydroxyethoxy-phenylthio-thymine (HEPT) series, was selected as the template due the structural similarity between the HEPT and DABO series. Figure 2 shows the structures of MKC-442 and **59**, the most potent NH-DABO derivative used in this study. All structures were constructed and fully geometry optimized at the AM1 semi-empirical level of theory in the SPARTAN′06 program [69].

#### 1.3.4. Construction and Optimization of the Protein-Ligand Complexes

The protein-ligand complexes were constructed based on the same co-crystallized structure (MKC-442/HIV-1 RT, PDB ID: 1RT1) [67] used in the ligands construction. This complex, obtained by X-ray crystallography with a 2.55 Å resolution, was employed with success in various molecular modeling studies, such as molecular docking, molecular dynamics, and 3D-QSAR [70,71,72,73,74,75].

In the construction of the protein-ligand complexes, each optimized DABO was manually docked in the NNBS of the reference complex, by root mean square (RMS) overlapping with the MKC-442, using the HyperChem 7.5 program [76]. The MKC-442 (superimposed on the ligand) and the water molecules (present in the original structure) were excluded from the protein-ligand complexes, the hydrogen atoms were added and the basic (Lys and Arg) and acid (Asp and Glu) amino acids were ionized.

The protein-ligand complexes were geometry optimized, with the goal of minimizing the possible unfavorable van der Waals contacts, in a three-step procedure as follows: (i) ligand; (ii) enzyme; and (iii) the entire complex. Using the steepest descent algorithm, it was carried out 1,000 optimization cycles or until to achieve a gradient lower than 0.01 kcal/mol Å. Subsequently, using the conjugate gradient algorithm, the resultant geometries were submitted to 1,000 optimization cycles or until the same convergence criterion. These calculations were performed in vacuum and without any geometric restrictions, employing the Tripos force-field in the SYBYL v.7.2 program [77].

#### 1.3.5. Molecular Dynamic Simulation of the Protein-Ligand Complexes

Before the molecular dynamics simulation (MDS) step, structures of the complexes were subjected to a new stage of geometry optimization, using the Gromos87 force field [78], available in the GROMACS program [79]. The topology of the ligands, needed in the MDS step, was built on the PRODRG server [80,81]. The atomic partial charges, calculated in this server, were replaced by those calculated by the semi-empirical method AM1 derived from the molecular electrostatic potential in the SPARTAN'06 program [69]. The MDS step was carried out, using the Gromos87 force field [78], which was chosen because of the facility in building a large number of ligands topology on the Dundee PRODRG server [80,81].

Finally, the complexes were submitted to the MDS in conditions of constant temperature (310 K) and pressure (1 atm), with the cutoff of 9 Å to the long-range electrostatic interactions and for non-bonded ones, using the Particle-Mesh Ewald (PME) method [82]. The SHAKE algorithm was used to keep fixed the length of the bonds [83]. The time of integration was 1 fs. Following the initial speed according to the Maxwell-Boltzmann distribution, the simulations were carried out in a tentative time of 1,000 ps (1 ns). However, as the interaction energies in 100 ps became nearly constant, this was the standard time used for the collection of energy values.

#### 1.3.6. Residue-Ligand Interaction Energies of the Complexes

**Figure 3:**
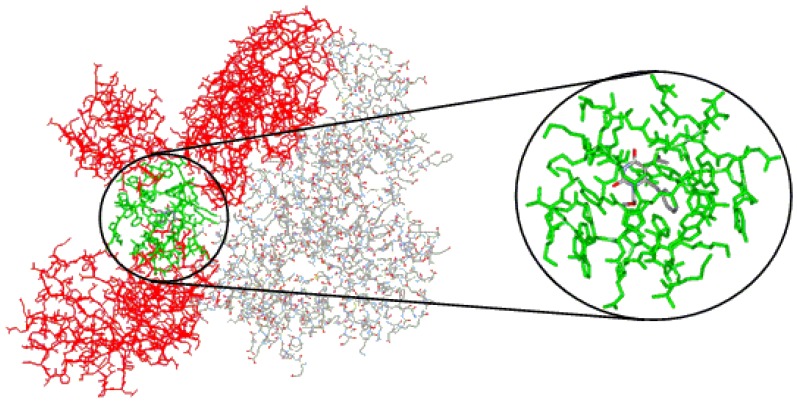
Schematic representation of the MKC-442/RT complex (PDB code 1RT1). At left, it is shown the RT structure with the p66 (colored in red) and p51 (colored by element) subunits, where the circle defines the radius of 10 Å from the ligand (MKC-442). At right, this region is shown in close up with the enclosed enzyme residues (colored in green) and the ligand (colored by element).

The descriptors (independent variables) in this new ReLIE-3D-QSAR method are the steric and electrostatic interaction energies of the protein-ligand complexes (*per residue*) simulated by molecular dynamics. Therefore, the steric and electrostatic interaction energies between each one of the 74 ligands and the amino acid residues of the enzyme, included within a 10 Å radius around the ligand (Figure 3), comprising 53 amino acids, were retrieved from the MD simulation step performed as described earlier in the GROMACS program, which employs the Lennard-Jones and Coulomb potentials to calculate the steric and electrostatic interaction energies, respectively [84].

This procedure was adopted considering that the protein-ligand interactions that contribute most significantly to the variation in inhibitory response occur with specific residues of the enzyme, near the binding site [85,86]. It has analogy with the pruning approach developed by Tokarski and Hopfinger (1997) in 3D-QSAR studies [87], in which the energy terms connecting the protein-ligand complexes are calculated by the free energy force field (FEFF) method in reduced models of the complexes [48]. In 2011, our group has published RD-3D-QSAR models using the ReLIE approach applied to acetylcholinesterase inhibitors [88].

#### 1.3.7. Definition of the Independent Variables Databases

To assess the influence of the independent variables database (descriptors) on the predictive ability of the models to be generated, we tested four databases (**DBs**) (Table 2), varying the number, combination, and pre-treatment of descriptors, as follows.

**Table 2:** Summary of features of the four databases (**DB**) used in the ReLIE-3D-QSAR analysis.

DB	Features	Total number of descriptors
**DB-I**	Lennard-Jones (LJ) and Coulomb (C) energies calculated individually by residue	106 (53 LJ + 53 C)
**DB-II**	Sum of **DB-I** descriptors by residue	53 (53 LJ + C)
**DB-III**	**DB-I** + **DB-II**	159 (53 LJ + 53 C + 53 LJ+C)
**DB-IV**	**DB-I** pre-treatment by exclusion of energies columns with variance (<0.0001)	95 (42 LJ + 53 C)

(a) The first database, **DB-I**, corresponds to the original database, in which the descriptors are the steric and electrostatic interaction energies calculated individually by residue, using the Lennard-Jones (LJ) and the Coulomb (C) potentials, respectively. Considering that the protein-ligand complex models contain 53 amino acids, the total number of descriptors (53 LJ and 53 C) in **DB-I** is equal to 106.

(b) In the second database, **DB-II**, the descriptors correspond to the sum of the Lennard-Jones and the Coulomb energies by residue, with a total of 53 descriptors (LJ + C).

(c) The third database, **DB-III**, is the combination of the two previous databases, with a total of 159 descriptors (53 LJ, 53 C and 53 C + LJ). 

(d) The fourth database, **DB-IV**, is the **DB-I** (106 descriptors) after pre-treatment, in which the descriptors with variance values lower than 0.0001 were excluded, with a total of 95 descriptors. This pre-treatment was done in order to exclude variables that, probably, do not contribute to the explanation of the biological response change.

#### 1.3.8. Construction of the ReLIE-3D-QSAR Equations

To obtain the ReLIE-3D-QSAR equations, the four databases of independent variables, along with the values of biological activity (pIC_50_), were submitted individually to the Wolf program [53]. In this program, the independent variables (interaction energies) were confronted with the dependent variable (pIC_50_) through the GFA-PLS method [36,53].

The first step in the Wolf program was the generation of an initial population of 100 equations, each one containing four descriptors selected randomly from the DB. We tested various combinations of options within the GFA-PLS technique, setting up 100% mutation probability after each crossover operation and 10,000 and 50,000 crossover operations. The equations coefficients were calculated by PLS regression analysis, using 3, 4, 5 and 6 principal components. The algorithm that adjusts the number of independent variables in the models, smoothing-factor, was adjusted from 0.2 to 0.6 (using increment of 0.1). The combination of these options was undertaken in order to obtain models containing five to twelve independent variables (terms) and resulted in 40 sets of options, which were tested for each one of the four databases (**DB-I**, **DB-II**, **DB-III** and **DB-IV**), resulting in a total of 200 equations of ReLIE-3D-QSAR to be analyzed.

#### 1.3.9. Internal Validation of the ReLIE-3D-QSAR Models

The ten best models of each GFA-PLS analysis were classified according to the values of Friedman’s lack-of-fit (LOF) score [53], which is the penalized least square error (LSE) measure; *i.e.*, when two equations have the same LSE, the one which has the lowest number of terms (independent variables) has the lowest LOF and is the best equation [53]. Subsequently, the best equations were submitted to the leave-one-out cross-validation (LOO_cv_) technique, giving the cross-validated r^2^ value (q^2^).

In order to avoid model overfitting, it is assumed that the maximum number of terms must be in the ratio of at least five compounds in the database for each term in the equation [72,89]. Thus, the maximum number of terms has been obtained by dividing the total number of compounds from the training set (N = 59) by five, which results in 11.8 terms. Therefore, the models with twelve or more variables were not considered for further analysis.

#### 1.3.10. External Validation of the ReLIE-3D-QSAR Models

The significance and utility of 3D-QSAR models is generally checked by predicting the activity values of a set of compounds, named test set, which are not included in model development. The 15 molecules from the test set (**60**–**74**) were constructed and minimized as described for the training set ones. They were aligned with the most potent derivative (**59**) using atom-based RMS fitting.

#### 1.3.11. Selecting the Best Model

The various models obtained after the cross-validation process were ordered by the number of terms (which ranged from 5 to 12 independent variables) contained in each equation, considering for the qualitative analysis those with the highest values of q^2^ and r^2^, the lowest values of SE_cv_ and SEE, and a smaller number of outliers [90]. To compare models with different number of terms, the values of q^2^ were transformed into adjusted q^2^ [31], according to Equation 1.


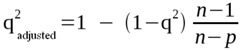
(1)

In Equation 1, **q^2^** represents the r^2^ value after cross-validation, **n** is the number of compounds from the training set and **p** is the number of variables (terms) in the model.

#### 1.3.12. Analysis of the Cross-Correlation Matrix of Residues

The cross-correlation matrix between the residues of the best selected models was used to analyze the correlation coefficients (r). The residual values represent the difference between the experimental (pIC_50Obs_) and the calculated (pIC_50Pred_) biological activity values.

According to Rogers (1996) [54], it is expected that equivalent models have similar distributions of residues, and different models show patterns of residues not correlated. Therefore, this kind of analysis is a valuable tool for the determination of a subset of distinct models in a number of good models obtained in GFA-PLS analysis, eliminating models with the same kind of structure-activity information [43], and justifying the importance of the correlated models exclusion [55,56,57,60,61].

#### 1.3.13. Analysis of the Cross-Correlation Matrix of Descriptors

In addition, the cross-correlation matrix between the independent variables (descriptors) of the best selected models was used to analyze the correlation coefficients (r), in order to determine if two or more variables highly correlated appear simultaneously in the same model [28,55,56,57,60,61]. With this approach, models showing redundant information could be excluded [28,31,61].

## 2. Results and Discussion

### 2.1. Analysis of the Reduced Models of the Protein-Ligand Complexes

**Figure 4:**
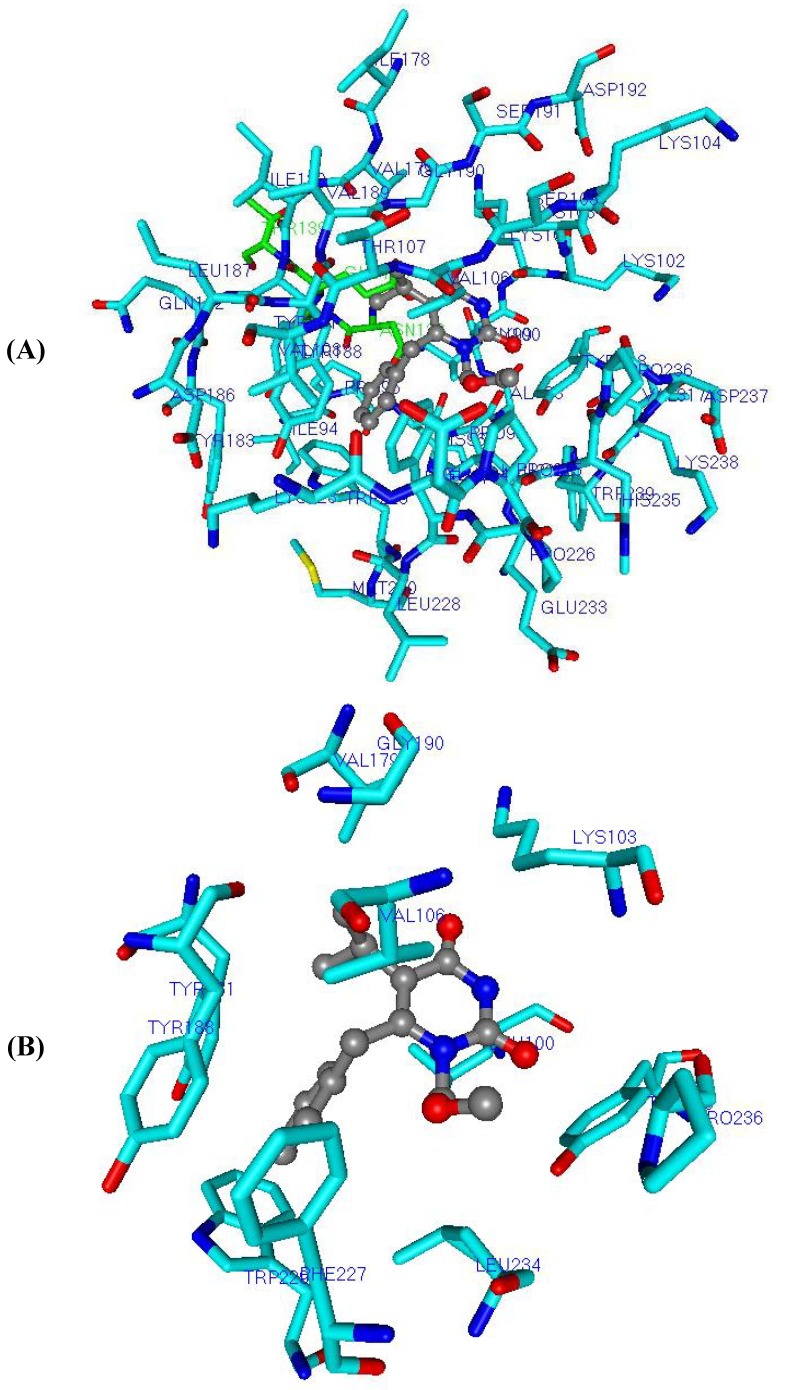
(**A**) Reduced model complex of the RT of HIV-1 (stick model, carbon atoms of the p66 subunit in light blue and p51 in green) showing all 53 residues included in the radius of 10 Å from the inhibitor MKC-442 (ball-and-stick model, carbon atoms in gray). (**B**) Close view of the reduced model complex, showing only the residues included in the radius of 5 Å.

As stated before, in order to calculate the steric and electrostatic interaction energies of each of the 74 protein-ligand complexes, we considered only the 53 residues in a radius of 10 Å (reduced model complex), defined from the ligand, as follows, in accordance with the subunit to which they belong (Figure 4A):

(a) Subunit p66: Ile94, Pro95, His96, Pro97, Ala98, Gly99, Leu100, Lys101, Lys102, Lys103, Lys104, Ser105, Val106, Thr107, Val108, Ile178, Val179, Ile180, Tyr181, Gln182, Tyr183, Asp186 (catalytic), Leu187, Tyr188, Val189, Gly190, Ser191, Asp192, His198, Lys223, Glu224, Pro225, Pro226, Phe227, Leu228, Trp229, Met230, Tyr232, Glu233, Leu234, His235, Pro236, Asp237, Lys238, Trp239, Thr240, Tyr317, Tyr318, and Asp319;

(b) Subunit p51: Asn136, Asn137, Glu138, and Thr139.

Considering the RT catalytic triad (Asp110, Asp185, and Asp186), Asp186 is the only residue included in the reduced model complex. In addition, residues Leu100, Lys101, Lys103, Val106, Val108, Tyr181, Tyr188, Gly190, Pro225, and Phe227, included in the reduced model complex, correspond to NNRTI-associated positions of frequent mutation [4,22,25,67,91].

To facilitate the discussion of results about the spatial location of the residues in the reduced model complex, we consider also a smaller radius of 5 Å from the ligand, which includes only the following residues (Figure 4B): Leu100, Lys103, Val106, Val179, Tyr181, Tyr188, Gly190, Phe227, Trp229, Leu234, Pro236, and Tyr318.

### 2.2. Overall Analysis of the Best Equations of Databases I to IV

The best ReLIE-3D-QSAR equation from each database studied (**DB-I**, **DB-II**, **DB-III** and **DB-IV**, Table 2) was selected considering the statistical values and the number of outliers, with the goal to select the most representative equation. The statistical indices are shown on Table 3 and the equations, on Table 4.

**Table 3:** Statistical results of the best equations of **DB-I** to **DB-IV**.

Equation (DB)	NTE ^a^	q^2^_adjus_^b^	q^2^^ c^	SE_CV _^d^	PC ^e^	r^2^^ f^	SEE ^g^	Outliers ^h^
**Eq.E (DB-I)**	10	0.660	0.713	0.420	4	0.822	0.500	3
**Eq.J (DB-II)**	10	0.606	0.667	0.460	6	0.766	0.600	3
**Eq.L (DB-III)**	7	0.594	0.636	0.480	3	0.723	1.180	4
**Eq.Q (DB-IV)**	9	0.616	0.669	0.458	6	0.764	0.700	5

^a^ Number of terms in the equation (NTE); ^b^ q^2^ adjusted; ^c^ r^2 ^after cross-validation (q^2^); ^d^ Standard deviation after cross-validation (SE_cv_); ^e^ Number of principal components (PC); ^f^ Quadratic correlation coefficient (r^2^); ^g^ Standard deviation of the estimate (SEE); ^h^ Number of outlier compounds in the data set.

**Table 4:** Descriptors selected in the best equations of **DB-I** to **DB-IV**.

**Eq.E**	**pIC50** = 4.853 + 22.417 **Ile94LJ** + 0.231 **Pro97LJ** − 0.153 **Lys101LJ**
**(DB-I)**	− 0.110 **Tyr181LJ** − 0.791 **Gln182C **− 0.122 **Tyr188LJ** + 0.323 **Ser191C**
	+ 0.043 **Pro226C** + 0.087 **His235LJ **− 56.813 **Asn137LJ**
**Eq.J**	**pIC50** = 6.802 + 0.101 **Gly99 **− 0.244 **Tyr183 **+ 1.202 **Leu187 **− 0.059 **Tyr188**
**(DB-II)**	+ 0.360 **Ser191 **+ 0.822 **Glu224 **− 0.028 **Phe227 **− 0.026 **Trp229**
	+ 0.061 **Asp237** + 1.437 **Thr139**
**Eq.L**	**pIC50** = 6.257 + 15.851 **Ile94LJ** − 0.118 **Tyr181LJ** + 0.101 **Pro225C**
**(DB-III)**	+ 3.525 **Glu224 **+ 0.062 **His235LJ** − 52.568 **Asn137LJ **− 0.008 **Glu138C**
**Eq.Q**	**pIC50** = 7.706 + 0.151 **Gly99C** + 1.162 **Leu187C** − 0.050 **Tyr188C**
**(DB-IV)**	+ 0.403 **Pro225C** − 0.238 **Pro226C** − 0.282 **Val179LJ**
	+ 0.124 **Lys103LJ** − 9.989 **Asn136LJ** − 0.074 **Phe227LJ**

Considering the four equations (Table 3 and Table 4), **Eq.E (DB-I)** was the best one, not only for the highest explanatory ability (high value of r^2^ and low value of SEE), but also for the greatest predictive ability, both internally (high value of q^2^ and low value of SE_CV_) (Table 3), in which the compounds of the training set have the lowest residual values, and externally, in which the compounds of the test set also showed the lowest residual values (Table 5). 

**Table 5:** Cross-correlation matrix among the residual values of Equations **E**, **J**, **L**, and **Q**.

	Eq.E	Eq.J	Eq.L	Eq.Q
**Eq.E**	1.000			
**Eq.J**	**0.559**	1.000		
**Eq.L**	0.514	0.434	1.000	
**Eq.Q**	0.289	0.278	0.474	1.000

The second best equation, **Eq.J **(**DB-II**), has the same number of terms (ten) and the same number of outliers (three) than **Eq.E**, however, it has less explanatory and predictive ability (Table 3). Equations **L** (**BD-III**) and **Q** (**BD-IV**), although more economical (showing only seven and nine terms, respectively), showed a higher number of outliers (*i.e.*, four and five, respectively). Moreover, in the case of **Eq.L**, the residual values of four outlier compounds (from the test set) are excessively high, making this equation the worst of all. Therefore, we can classify **Eq.E** and **Eq.J** as the two best equations and **Eq.L** and **Eq.Q** as the two worst.

In relation to the Lennard-Jones (LJ) and Coulomb (C) terms contribution (Table 4) on the structure-activity relationship (SAR), there is a greater prevalence of the LJ term in both the best (**Eq.E**, seven LJ and three C terms) and the worst equations (**Eq.L**, four LJ and two C terms), whereas in **Eq.Q**, there is a slight predominance of the C term (four LJ and five C terms). This analysis cannot be performed for **Eq.J**, because the contributions of LJ and C terms are not individualized, *i.e.* each term is the sum of the steric and electrostatic interaction energies.

Considering all the 53 amino acids contained in the reduced model complex (10 Å radius), 23 residues, namely **Ile94**, **Pro97**, **Gly99**, **Lys101**, **Lys103**, **Val179**, **Tyr181**, **Gln182**, **Tyr183**, **Leu187**, **Tyr188**, **Ser191**, **Glu224**, **Pro225**, **Pro226**, **Phe227**, **Trp229**, **His235**, **Asp237**, **Asn136**, **Asn137**, **Glu138**, and **Thr139**, appear more frequently in the best equations (**E**, **J**, **L**, and **Q**, Table 4). This indicates that these residues (~43%) are more important in the SAR than the others, independent of the kind of term contribution (*i.e.*, steric, electrostatic or combined) related to them.

Among these 23 residues, one (**Tyr188**) occurs in three equations (**Eq.E**, **Eq.J**, and **Eq.Q**), and eleven occur in two equations, namely **Ile94** (**Eq.E** and **Eq.L**), **Gly99** (**Eq.J** and **Eq.Q**), **Tyr181** (**Eq.E** and **Eq.L**), **Leu187** (**Eq.J **and **Eq.Q**), **Ser191** (**Eq.E** and **Eq.J**), **Glu224** (**Eq.J** and **Eq.L**), **Pro225** (**Eq.L** and **Eq.Q**), **Pro226** (**Eq.E** and **Eq.Q**), **Phe227** (**Eq.J** and **Eq.Q**), **His235** (**Eq.E** and **Eq.L**), and **Asn137** (**Eq.E** and **Eq.L**).

In the four equations (Table 4), at least one term is related to one of the amino acid residues of the p51 subunit: **Asn136** (term **Asn136LJ** of **Eq.Q**), **Asn137** (term **Asn137LJ** of **Eq.E** and **Eq.L**), **Glu138** (term **Glu138C** of **Eq.L**) and **Thr139** (term **Thr139** of **Eq.J**). As it will be discussed in details for **Eq.E**, this fact implies the importance of interchain interaction (p66-p51) in the equations that best describe the structure-activity relationship for this class of compounds.

Additionally, Table 5 shows the cross-correlation matrix between the residual values (pIC_50Obs_ − pIC_50Pred_) calculated for the training set compounds, using the four equations (**E**, **J**, **L**, and **Q**), so as to verify the correlation degree between these models. In such matrix, pairs of equivalent models may have correlated residual values (r close or equal to 1) and may represent the training set in a similar manner. Furthermore, pairs of distinct models have residual values not correlated (r < 0.7) [43]. Analyzing the data on Table 5, it is observed that, according to this statement, the models are not correlated (*i.e.*, they are distinct), as the highest correlation (r = 0.559) occurs between equations **E** and **J**, which are the two best models, while **Eq.Q** is the model that shows most divergence from the others. 

### 2.3. Analysis of the Best Equation of BD-I (Eq.E)

**Figure 5:**
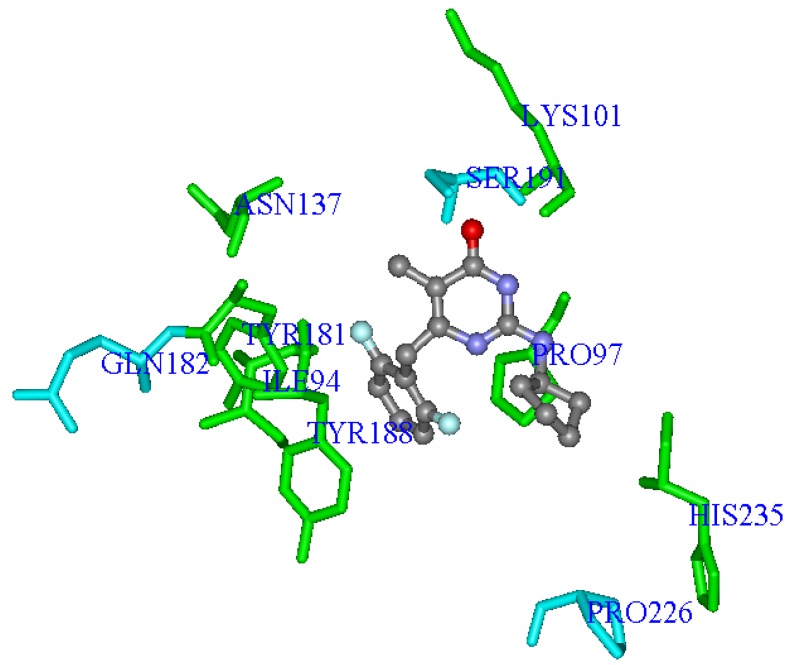
Three-dimensional graphic representation of **Eq.E** (Table 3 and Table 4, **DB-I**), using the most potent NH-DABO, compound **59** (stick-and-ball model colored by element), as example. The amino acids residues (stick model) colored in green (**Ile94**, **Pro97**, **Lys101**, **Tyr181**, **Tyr188**, **His235** and **Asn137**) represent Lennard-Jones contributions and those colored in light blue (**Gln182**, **Ser191** and **Pro226**) represent Coulomb contributions. The hydrogen atoms were omitted for better viewing.

In **Eq.E **(**BD-I**) (Table 3 and Table 4, Figure 5), each one of the 10 independent variables (steric and electrostatic interaction energies calculated by amino acid residue) is represented by the corresponding amino acid three letters code, followed by the LJ (Lennard-Jones) or C (Coulomb) designation, which indicates that the interaction refers to the steric or electrostatic contribution, respectively. Figure 5 shows the **Eq.E** three-dimensional graphic representation, using the most potent NH-DABO, compound **59**, as example.

It should be noted that residues **Lys101**, **Tyr181**, **Tyr188**, and **His235**, which are described in the literature as often making interactions with several NNRTIs, were selected in this equation [20,21,63,92,93].

In **Eq.E**, only three terms (**Gln182C**, **Ser191C**, and **Pro226C**) represent Coulomb contributions, while the other seven terms (**Ile94LJ**, **Pro97LJ**, **Lys101LJ**, **Tyr181LJ**, **Tyr188LJ**, **His235LJ**, and **Asn137LJ**) represent Lennard-Jones contributions. This result indicates a higher importance of the steric interaction than of the electrostatic one to the structure-activity relationship, corroborating the importance of the steric interaction in the hydrophobic cavity of NNBS as discussed by several authors [20,21,92,94]. In addition, the three electrostatic terms (**Gln182C**, **Ser191C**, and **Pro226C**) selected in this equation are related to residues that are located outside the radius of 5 Å, as defined previously, which might be justified because the electrostatic interaction has a larger range than the steric one.

The values of pIC_50_ calculated (predicted) by **Eq.E** are influenced by the magnitude and the signal (+ or −) of the coefficient for each term in the equation and by the signal of the interaction energy itself. As an example, the coefficient of the term **Tyr181LJ** is negative (−0.110) in **Eq.E**, therefore, the steric interaction energy between this residue **Tyr181** and a ligand should be negative for this energy term to contribute to increase the compound potency; if the interaction energy is positive, the term will help reduce the potency of the compound.

In contrast, the term **Ile94LJ** presents the positive coefficient (22.417) in **Eq.E**, therefore, the steric interaction energy between this residue **Ile94** and a ligand should be positive for this term of energy to increase the potency of the compound, if the interaction energy is negative, the term will reduce the potency of the compound. 

**Figure 6:**
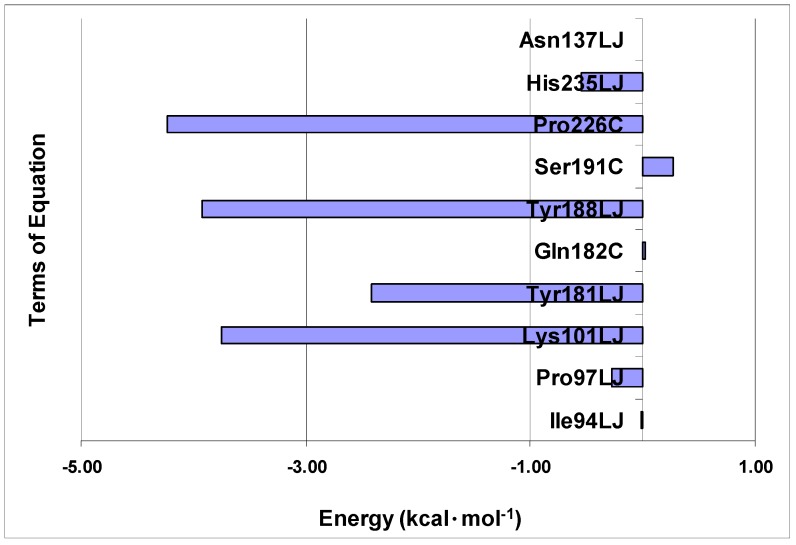
Average interaction energies (kcal·mol^−1^) of compounds **1**–**74** with each of the selected terms in accordance with **Eq.E (DB-I)**.

Figure 6 shows the average values of interaction energy (kcal·mol^−1^) of compounds **1**–**74** with the residues selected in **Eq.E**. Most inhibitors presents negative (or close to zero) interaction energies with most of the residues of the **Eq.E**, except with residue **Ser191**, in which the energy values are negative and of greater magnitude for the terms **Lys101LJ**, **Tyr181LJ**, **Tyr188LJ** and **Pro226C**, with an average energy of about −3.8 kcal·mol^−1^. The terms **Pro97LJ** and **His235LJ**, which also represent the negative values of energy, have an average energy of about −0.4 kcal·mol^−1^. Considering these six residues with the negative values of energy, the terms **Lys101LJ**, **Tyr181LJ**, **Tyr188LJ**, provide the coefficient of negative sign (**Eq.E**), to increase the potency, while the terms **Pro97LJ**, **Pro226C** and **His235LJ**, which have the coefficient of positive sign (**Eq.E**), contribute to decrease the potency. 

The terms **Ile94LJ**, **Gln182C** and **Asn137LJ**, providing average energy of interaction close to zero, are those with the highest values (modular) coefficients, which allows them to balance the weight in relation to other terms in **Eq.E**. It is also interesting to note that these three terms are related to residues that may be found outside the radius of 5 Å, which may also explain the lower interaction energy (modular) we were able to calculate.

Finally, the term **Ser191C** (**Eq.E**) is the only one that shows average values of positive interaction, which correspond to a repulsive electrostatic interaction, *i.e.*, negative, with a value close to 0.3 kcal·mol^−1^. Curiously, this term contributes to the increase in the potency, since it presents positive coefficient in **Eq.E**. Other authors have reported positive steric and electrostatic interaction energies for other systems [92].

Concerning the frequently mutated residues in RT related to NNRTIs, three of them were selected in **Eq.E** related to the terms **Lys101LJ**, **Tyr181LJ** and **Tyr188LJ**. As these three terms have negative steric interaction energy values and also negative coefficients in **Eq.E**, all contribute to increase the potency. 

**Figure 7:**
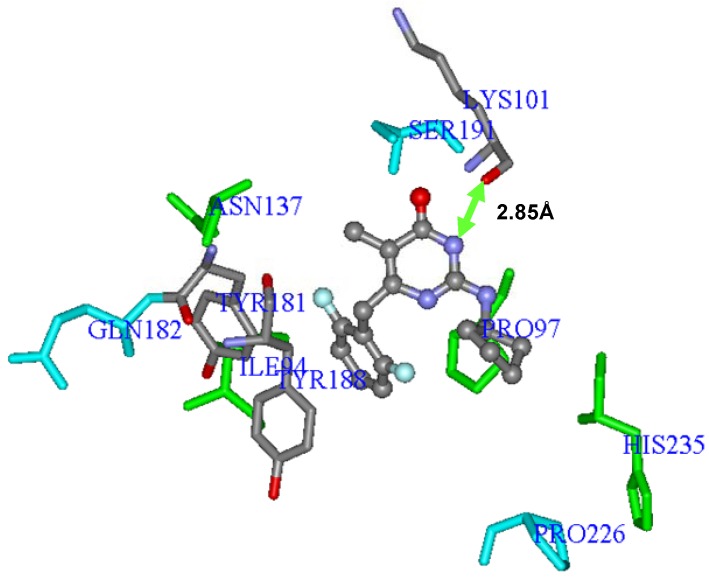
Three-dimensional graphic representation of **Eq.E** (Table 3 and Table 4, **DB-I**), using the most potent NH-DABO, compound **59** (stick-and-ball model colored by element), highlighting the frequently mutated residues **Lys101**, **Tyr181**, and **Tyr188** (stick model colored by element). The arrow indicates a possible hydrogen interaction between **59** and **Lys101**. The residues (stick model) colored in green (**Ile94**, **Pro97**, **His235**, and **Asn137**) and by element (**Lys101**, **Tyr181**, and **Tyr188**) represent Lennard-Jones contributions and those colored in light blue (**Gln182**, **Ser191**, and **Pro226**) represent Coulomb contributions. The hydrogen atoms were omitted for better viewing.

Interestingly, after the mutation of **Lys101Gly** into the wild-type RT, the oxygen atom of the amide group’s main chain of **Lys101** was capable of making hydrogen interaction (2.85 Å) with the -NH group of the 4-oxo-pyrimidine inhibitors, as shown in Figure 7 for compound **59**. Additionally, the protonated amine side chain of this residue belonging to subunit p66 was able to make ionic interaction with the carboxylate group of the side chain of **Glu28** (about 5 Å away) and **Glu138** (at around 6.5 Å away), both belonging to the p51 subunit, and responsible, therefore, for interchain interactions (p66-p51).

It’s curious that after the change into **Gly101**, the interaction between the residue and the hydrogen atom of the inhibitor could be maintained, since it involves the main chain's residue, which is not changed. However, the corresponding interchain interactions, which depend on the side chain of residue, were lost, affecting the composition of the NNBS (referring to the residue of the p51 subunit). The interchain interactions are important for the dimerization process of the RT and it is composed by residues from p66 and p51 [95].

In the best four equations, we have found at least one term that is related to an amino acid residue of the p51 subunit: **Asn136** (**Eq.Q**), **Asn137** (**Eq.E**), **Glu138** (**Eq.L**), and **Thr139** (**Eq.J**). Both **Asn136** and **Asn137** are highly conserved among the heterodimeric RTs, e.g., HIV-1, HIV-2 and simian immunodeficiency virus [95]. This fact points to a defined (but as yet unidentified) functional and/or structural role for these residues. The highly conserved **Asn136** is in close proximity to the NNRTI lipophilic pocket of HIV-1 RT. Site-directed mutagenesis has revealed that the catalytic activity of HIV-1 RT mutated at position **Asn136** is heavily compromised [95]. Only 0.07 to 2.1% of wild-type activity is retained, depending on the nature of the amino acid change at position **136 **[95].

Furthermore, the mutations **Tyr181Cys** and **Tyr188Leu** belong to a more common case, in which the exchange between the residue affects directly the protein-ligand interaction, and not, as in the previous case, indirectly. In both mutations there is a loss of the π-π-stacking interactions between the side chain aromatic ring of **Tyr181** and **Tyr188** with the inhibitors aromatic ring, reducing the affinity for the NNBS [92,94].

### 2.4. Analysis of Residual Values of the Best Equation of DB-I (Eq.E)

Table 6 shows the pIC_50_ (M) values observed (experimental) and predicted by **Eq.E** and their residual values (pIC_50Obs_ − pIC_50Pred_) for the training (**1**–**59**) and test (**60**–**74**) sets. 

**Table 6:** Values of pIC_50_ observed, predicted and residual values (pIC_50Obs_ − pIC_50Pred_) for the training (**1**–**59**) and test (**60**–**74**) sets according to the **Eq.E (DB-I)**.

#	pIC_50Obs_	pIC_50Pred_	Res			#	pIC_50Obs_	pIC_50Pred_	Res
**1**	4.23	4.23	0.00			**38**	6.10	5.95	0.15
**2**	4.31	4.34	−0.03			**39**	6.10	6.09	0.01
**3**	4.35	4.30	0.05			**40**	6.10	5.96	0.14
**4**	4.59	5.41	−0.82			**41**	6.10	6.66	−0.56
**5**	4.77	4.97	−0.20			**42**	6.22	6.27	−0.05
**6**	4.79	5.35	−0.56			**43**	6.22	6.47	−0.25
**7**	4.83	5.14	−0.31			**44**	6.22	5.87	0.35
**8**	4.83	4.94	−0.11			**45**	6.30	6.04	0.26
**9**	5.02	4.99	0.03			**46**	6.40	6.15	0.25
**10**	5.07	5.37	−0.30			**47**	6.70	6.73	−0.03
**11**	5.09	4.94	0.15			**48**	6.70	6.42	0.28
**12**	5.27	5.24	0.03			**49**	6.70	6.56	0.14
**13**	5.31	5.88	−0.57			**50**	6.92	6.48	0.44
**14**	5.31	5.03	0.28			**51**	7.00	6.89	0.11
**15**	5.32	5.79	−0.47			**52**	7.00	7.25	−0.25
**16**	5.34	5.19	0.15			**53**	7.05	6.88	0.17
**17**	5.37	5.67	−0.30			**54**	7.05	6.98	0.07
**18**	5.42	5.24	0.18			**55**	7.10	6.45	0.65
**19**	5.44	5.38	0.06			**56**	7.10	6.86	0.24
**20**	5.47	5.42	0.05			**57**	7.15	6.61	0.54
**21**	5.49	5.60	−0.11			**58**	7.30	7.51	−0.21
**22**	5.52	5.59	−0.07			**59**	7.52	6.68	0.84
**23**	5.52	6.04	−0.52			**60**	4.35	5.18	−0.83
**24**	5.52	5.57	−0.05			**61**	4.48	5.01	−0.53
**25**	5.54	5.53	0.01			**62**	5.27	6.03	−0.76
**26**	5.55	5.16	0.39			**63**	5.47	5.88	−0.41
**27**	5.59	5.61	−0.02			**64**	5.59	5.60	−0.01
**28**	5.60	6.00	−0.40			**65**	5.60	5.08	0.52
**29**	5.60	5.87	−0.27			**66**	5.62	6.02	−0.40
**30**	5.62	5.23	0.39			**67**	5.74	4.68	1.06
**31**	5.66	5.38	0.28			**68**	5.92	5.13	0.79
**32**	5.72	5.40	0.32			**69**	6.22	8.52	−2.30
**33**	5.80	6.37	−0.57			**70**	6.40	5.28	1.12
**34**	5.89	4.23	−0.34			**71**	6.70	7.14	−0.44
**35**	5.94	4.34	0.60			**72**	7.05	6.92	0.13
**36**	5.94	6.50	−0.56			**73**	7.15	6.35	0.80
**37**	5.96	5.75	0.21			**74**	7.30	7.06	0.24

**Figure 8:**
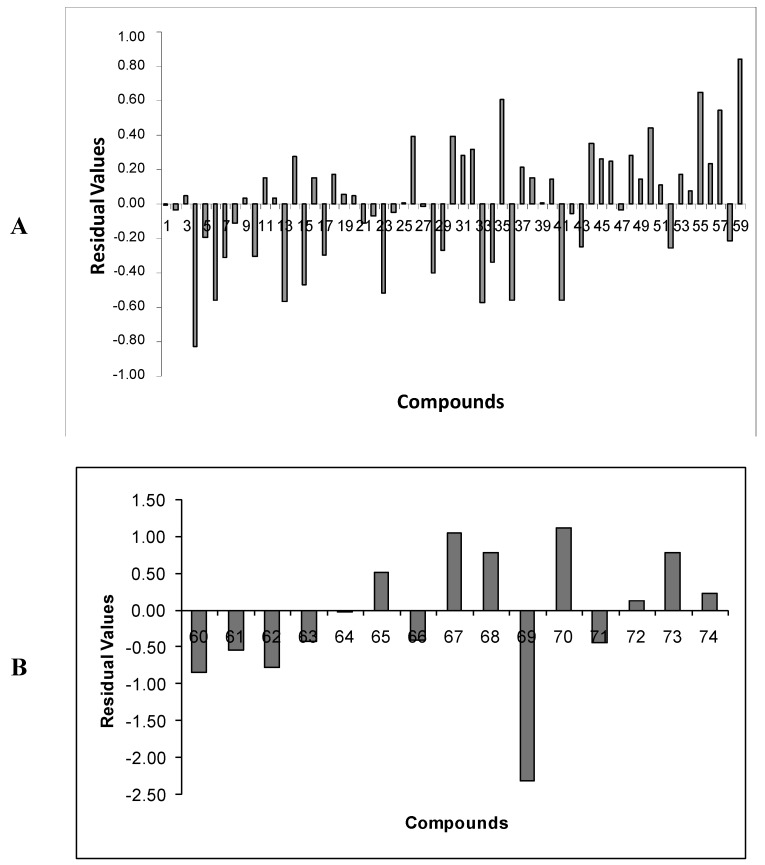
Residual values of compounds of the (A) training (**1–59**) and (B) test (**60**–**74**) sets according to the **Eq.E (DB-I)**.

For the training set (**1**–**59**), Table 6 shows that 81% of the residual values of compounds were lower than 0.50 (in modular values). Moreover, no compound of the training set is classified as an outlier, since none presented residual values greater than twice the standard error of estimate of the **Eq.E** (2 × 0.500 = 1.00). This shows an excellent internal predictive ability of the model. Figure 8A shows the residual values of the training set compounds as a graphic bar.

For the test set compounds (**60**–**74**), 40% have residual values lower than 0.50, while three compounds are considered outliers (**67**, **69** and **70**). Figure 8B shows residual values of the test set compounds as a graphic bar. It is important to emphasize that the residual values from both training and test sets showed random variations along the predicted potency. This means that the model is not biased to a higher or lower value of activity.

### 2.5. Analysis of Outliers of Eq.E (DB-I)

As mentioned earlier, with relation to **Eq.E**, three outliers were identified (**67**, **69**, and **70**), all of them from the test set (Table 1). Compounds **67** (residue = 1.06) and **70** (residue = 1.12) have predicted potencies lower than the experimental ones, while the compound **69** (residue = −2.30) has a predicted potency higher than the experimental one.

In the descriptors (interaction energies) selected in **Eq.E**, the terms which have more variation are **Tyr181LJ** and **Gln182C** related to steric and electrostatic contributions, respectively. For **Tyr181LJ**, the energy values obtained were: −2.540 kcal·mol^−1^ for compound **67**, −7.517 kcal·mol^−1^ for compound **69** and 1.620 kcal·mol^−1^ for compound **70**. This term has a negative coefficient (−0.110) in **Eq.E**, increasing the potency, something that may justify the higher potency predicted for **69**, since the aromatic ring of residue **Tyr181** makes hydrophobic π-π-stacking interactions with the aromatic rings of NNRTIs, as described above. However, it remains unclear why similar compounds containing the nitro substituent in *para* (**30**) or *meta* (**43**) position (Table 1) are not outliers, while **69** (*ortho*-nitro) is an outlier.

For **Gln182C**, which also has a negative coefficient (−0.791) in **Eq.E**, increasing the potency, the behavior seems similar to the previous term, but with less intensity, since the energy values obtained are as follows: 0.188 kcal·mol^−1^ (**67**), −0.606 kcal·mol^−1^ (**69**), and 0.097 kcal·mol^−1^ (**70**).

### 2.6. Analysis of MKC-442 According to Eq.E (DB-I)

The potency of the compound MKC-442, which belongs to the class of HEPTs, was predicted by **Eq.E**, as an additional external validation test, since this compound is not part of the database of DABOs. This was done because this compound was used as template in the construction and alignment of the DABOs in the NNBS (X-ray structure PDB 1RT1).

Thus, according to **Eq.E**, the compound MKC-442 has a predicted potency lower than the experimental one (pIC_50_ = 6.68 M), since the value of IC_50Obs_ for this compound is 0.04 mM [64], *i.e.*, pIC_50Obs_ = 7.40 M. Consequently, the residual value is equal to −0.72, which is less than twice the SEE of **Eq.E**, indicating that the compound was well predicted.

### 2.7. Analysis of the Cross-Correlation Matrix among the Eq.E Descriptors (DB-I)

The analysis of the cross-correlation matrix between the terms of **Eq.E** (Table 7) shows that there is no significant correlation between the various descriptors (steric and electrostatic interaction energies), since there was no value of r (linear correlation coefficient) higher than 0.70 (in modular value) [31]. This shows that each descriptor brings unique information to the model, therefore, there is not redundant information in this equation. The highest correlation is found between the terms **Tyr181LJ** and **Tyr188LJ** (r = 0.543), probably due to the spatial proximity between these residues in the corresponding NNBS and due to the same type of amino acid (Tyr) being involved.

**Table 7:** Cross-correlation matrix among the **Eq.E** descriptors.

	Ile94 LJ	Pro97 LJ	Lys101 LJ	Tyr181 LJ	Gln182 C	Tyr188 LJ	Ser191 C	Pro226 C	His235 LJ	Asn137 LJ
**Ile94** **LJ**	1.000									
**Pro97** **LJ**	−0.126	1.000								
**Lys101** **LJ**	0.155	−0.122	1.000							
**Tyr181** **LJ**	0.354	0.039	−0.131	1.000						
**Gln182** **C**	0.238	−0.107	0.116	−0.073	1.000					
**Tyr188** **LJ**	0.176	0.169	−0.212	**0.543**	−0.243	1.000				
**Ser191** **C**	0.086	0.001	0.260	0.194	0.043	0.166	1.000			
**Pro226** **C**	0.204	−0.083	0.214	0.200	0.056	0.029	0.313	1.000		
**His235** **LJ**	0.080	0.131	−0.087	−0.030	−0.203	−0.037	−0.320	−0.214	1.000	
**Asn137** **LJ**	0.435	−0.011	−0.099	0.036	0.216	0.012	−0.055	−0.012	0.135	1.000

### 2.8. Comparison of CoMFA (RI-3D-QSAR) and ReLIE (RD-3D-QSAR) Models

Recently, we reported a CoMFA (RI-3D-QSAR) model [62], using this same series of DABO derivatives. In short, the best CoMFA model was built with PM3 charges, default cutoff of 30 kcal·mol^-1^ for both steric and electrostatic fields, *sp*^3^ carbon atom with +1 charge as the probe atom and the grid spacing of 2.0 Å. This model showed good internal consistency in terms of r^2^ and SEE and good predictive ability (q^2^ = 0.691). In this study, the alignments, the partial atomic charges, the cutoff, and the probe atoms had little influence on the resulting statistical values and, consequently, on the models.

The most important structural conclusions from the CoMFA study were the restriction in the volume of the substituent at position **C2** of the 4-oxopyrimidine ring (Table 1), more bulky substituents in position **C5** and the presence of electron-rich groups in the position **C6 **of the aromatic ring, which increase biological activity, making these areas important sites for future structural changes. 

The pharmacoforic hypothesis proposed in the ReLIE study was based on MKC-442 conformation and orientation in the NNBS. It was validated by the good statistical results obtained. The best model, **Eq.E** (**DB-I**), shows q^2^ = 0.660, and the external predictive ability was evaluated using a test set of 15 compounds, the same used in the CoMFA.

Although the CoMFA model is little more predictive than the ReLIE model, the incorporation of the X-ray data of protein-ligand complex in the ReLIE studies provided a more detailed interpretation of the contour maps of CoMFA, leading also to better understanding of the interaction of the complex.

## 3. Conclusions

In this work, we built and evaluated the first residue-ligand interaction energy (ReLIE) receptor-dependent 3D-QSAR model of a series of S- and NH-DABOs as HIV-1 reverse transcriptase non-nucleoside inhibitors, where the descriptors are the steric and electrostatic interaction energies between ligands and residues from the protein-ligand complexes simulated by molecular dynamics.

The pharmacoforic hypothesis, based on MKC-442 conformation and orientation in the NNBS, was validated by the good statistical results obtained. In the four best equations, at least one term is related to one of the four amino acid residues of the p51 subunit: **Asn136**, **Asn137**, **Glu138**, and **Thr139**. This fact implies the importance of interchain interaction (p66-p51) in the equations that best describe the structure-activity relationship for this class of compounds. The best model, **Eq.E** (**DB-I**), shows q^2^ = 0.660, SE_cv_ = 0.500, PC = 6, r^2 ^= 0.930, and SEE = 0.226, and the external predictive ability was evaluated using a test set of 15 compounds. The model interpretation was consistent with the crystallographic data and highlighted important amino acids that interact with DABOs, which are **Ile94**, **Pro97**, **Lys101**, **Tyr181**, **Gln182**, **Tyr188**, **Ser191**, **Pro226, **and **His235.**

The steric interaction energy descriptor has more prevalence than the electrostatic one, since it is more present in the best equations for each **DB**, highlighting the importance of hydrophobicity in the SAR of this series of inhibitors.

Comparing this work with a recent CoMFA (RI-3D-QSAR) study published by our group using the same set of compounds used here, we find that the ReLIE (RD-3D-QSAR) study provides much more valuable information than CoMFA. This information can be used in structure-activity relationship of this class of compounds, and the equations can be used for the prediction of other compounds that belong to the same class of DABOs.

Also, given the importance of the conserved **Asn136** and **Asn137**, these residues therefore could become an attractive target for the design of novel NNRTIs with improved potency and increased ability to avoid development of drug-resistant viruses.
